# Photoactivated Polymersome Nanomotors: Traversing Biological Barriers

**DOI:** 10.1002/anie.202003748

**Published:** 2020-07-27

**Authors:** Jingxin Shao, Shoupeng Cao, David S. Williams, Loai K. E. A. Abdelmohsen, Jan C. M. van Hest

**Affiliations:** ^1^ Bio-Organic Chemistry Institute of Complex Molecular Systems Department of Biomedical Engineering Eindhoven University of Technology, Helix (STO 3.41) P. O. Box 513 5600 MB Eindhoven The Netherlands; ^2^ Department of Chemistry College of Science Swansea University Swansea SA2 8PP UK

**Keywords:** intracellular delivery, nanomotors, photothermal effect, pH-sensitive polymer, polymersomes

## Abstract

Synthetic nanomotors are appealing delivery vehicles for the dynamic transport of functional cargo. Their translation toward biological applications is limited owing to the use of non‐degradable components. Furthermore, size has been an impediment owing to the importance of achieving nanoscale (ca. 100 nm) dimensions, as opposed to microscale examples that are prevalent. Herein, we present a hybrid nanomotor that can be activated by near‐infrared (NIR)‐irradiation for the triggered delivery of internal cargo and facilitated transport of external agents to the cell. Utilizing biodegradable poly(ethylene glycol)‐*b*‐poly(d,l‐lactide) (PEG‐PDLLA) block copolymers, with the two blocks connected via a pH sensitive imine bond, we generate nanoscopic polymersomes that are then modified with a hemispherical gold nanocoat. This Janus morphology allows such hybrid polymersomes to undergoing photothermal motility in response to thermal gradients generated by plasmonic absorbance of NIR irradiation, with velocities ranging up to 6.2±1.10 μm s^−1^. These polymersome nanomotors (PNMs) are capable of traversing cellular membranes allowing intracellular delivery of molecular and macromolecular cargo.

## Graphical abstract

**Delivery driver**: Photo‐activated polymersome nanomotors (PNMs) composed of a biodegradable polymersome system coated with a hemisphere gold layer were utilized for intracellular delivery of molecular cargo via the assistance of a NIR laser. The active penetration of the cell membrane by the nanomotors allowed both encapsulated payloads and surrounding cargo to be delivered into cells.

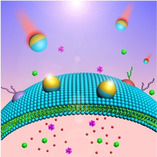

## Introduction

Autonomously propelled micro‐ and nano‐motors are devices that can convert various sources of energy into mechanical motion.^1^ As energy transducer, these miniaturized machines are powered through local chemical reactions and/or exogenous stimuli, such as electric/magnetic/ultrasound field and light.^2^ The development of motile vehicles, with inorganic or enzyme‐driven motors, has received much recent attention.^3^ Such systems focus on fuel‐driven and chemotactic behavior. Although chemically driven motility is a highly beneficial property, the ability to power motile systems using an external stimulus is often more advantageous when biomedical applications are envisaged, such as in the case of targeted theranostics. Elegant research has been published in which micro/nanomotors powered by physical stimuli have been demonstrated to perform complex tasks ranging from environmental remediation to therapeutic applications—although often biodegradability of the motor systems was not taken into account in the design.^4^ Furthermore, one important parameter that limits the use of synthetic motors in biomedicine‐related applications is size. Although examples of micron‐sized motors are numerous, one of the core challenges in this area is to miniaturize such technologies to the nanoscale. For example, intracellular delivery using micron‐sized particles presents a challenge to areas of research such as cell‐based therapies, diagnostics/analysis, and regenerative medicine.^5^

The rationale behind using synthetic motor systems as cellular delivery vehicles lies in the fact that motors can not only be guided to the right tissue type, but can also exert a force on the cell membrane to effectively transport the desired cargo to the intracellular domain. The cell membrane, as a robust biological barrier, is designed to deny passage to the vast proportion of extrinsic matter—especially macromolecular species, which undermines the efficient delivery of synthetic biofunctional materials.^6^ Tremendous efforts have been made to resolving this issue, largely involving either nanocarrier‐mediated or membrane‐disruption approaches.^7^ Carrier‐mediated strategies are mainly focused on encapsulation of molecular cargoes, followed by intracellular delivery via an endocytotic pathway.^8^ An apparent downside of this approach is the slow kinetics of uptake that could increase the risk of drug leakage during transportation. Efficient (and fast) delivery of macromolecular cargoes across the plasma membrane therefore remains a challenge.^6^ In this regard, the membrane disruption approach was presented as an effective pathway to address this issue; however, examples of nanoparticles that can undertake such a process are extremely limited—creating ample opportunity for exploration of non‐Brownian motor‐mediated active transport.

The membrane disruption approach takes advantage of physical processes by which the cell membrane is locally disrupted in order to rapidly introduce cargoes.^5^ External stimuli that can be used to induce such an effect include temperature,^9^ magnetic fields,^10^ electric field,^11^ ultrasound,^12^ or light.^13^ Gold‐based nanomaterials that display a photothermal effect when exposed to NIR light have received increasing attention.^14^ As demonstrated by pioneering research, NIR lasers are capable of deep tissue penetration.^15^ As such, they can remotely activate photothermal agents for therapeutic applications.^16^ and provide a driving force for active cargo transport^17^ by photomechanically disrupting the cell membrane.^18^ Compared to passive drug carriers, nanomotors with autonomous propulsion could enhance tissue binding and penetration, leading to prolonged retention of cargo in targeted sites.^19^

**Figure 1:**
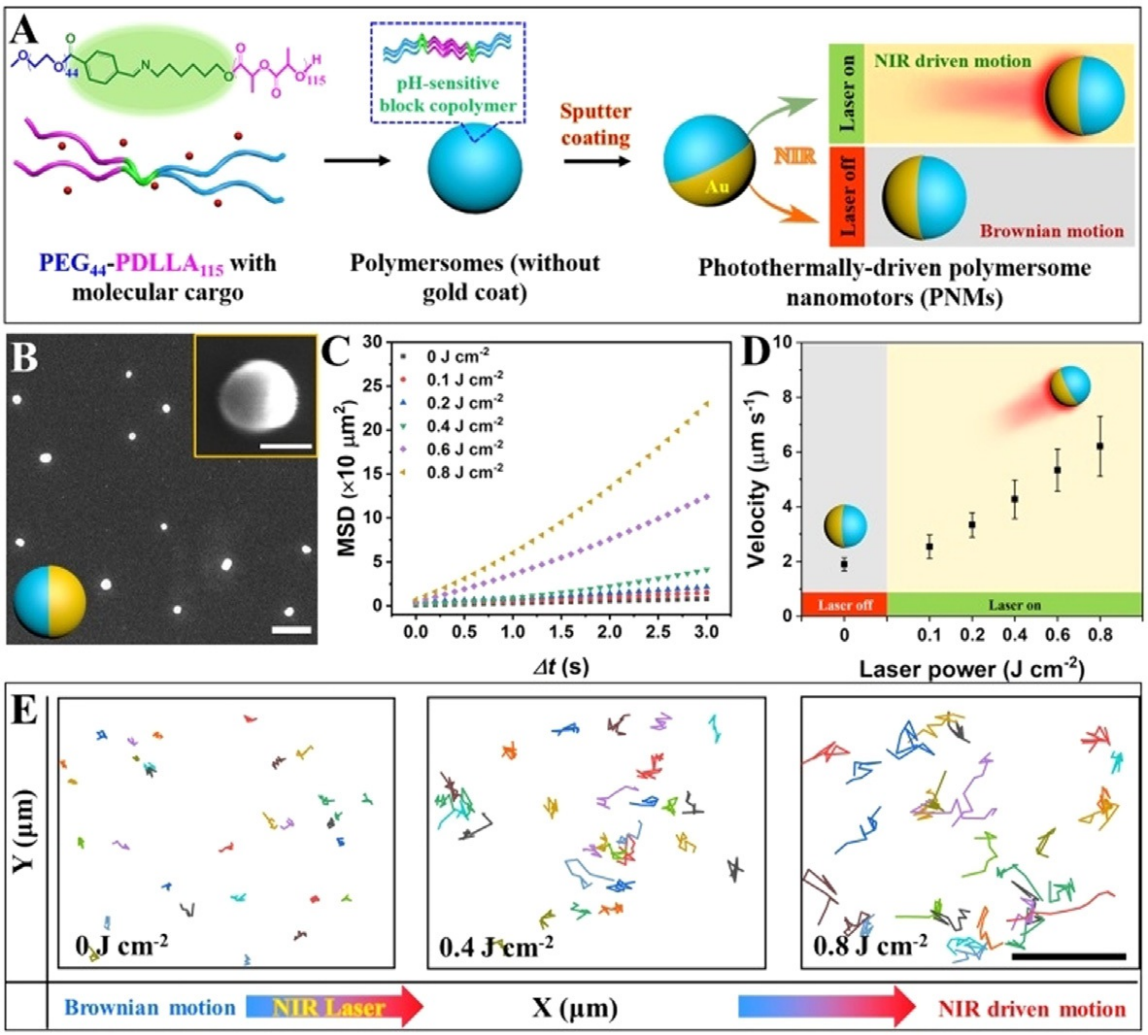
Preparation and characterization of photo‐activated, photothermally driven polymersome nanomotors (PNMs). A) Design strategy of PNMs. B) SEM image of PNMs (scale bar=1 μm/insert scale=100 nm). C) Mean square displacement (MSD) curves of motile PNMs at different laser power. D) Velocities of PNMs under different incident laser power. Inset image is the schematic overview of the transformation of PNMs from Brownian to non‐Brownian active transporters upon NIR irradiation. E) The trajectory pathways of PNMs as a function of the incident laser power (scale bar=50 μm).

To make the application of synthetic motors in biomedicine more feasible we report the development of nanosized and biodegradable nanomotors based on Au‐coated polymersomes, which display NIR‐activated intracellular transportation via membrane disruption. Such photothermally driven polymersome nanomotors (PNMs) can be used for the controlled delivery of biofunctional cargoes (Figure 1 A). Polymeric vesicles (polymersomes) comprising amphiphilic block copolymers are versatile compartments that have been employed as nanocarriers for a wide range of cargoes.^20^ Hydrophilic and hydrophobic cargoes can be both sequestered by polymersomes within distinct locations (i.e. aqueous core and non‐aqueous membrane). Furthermore, the chemical nature of polymersomes can be tailored to control, for example, its degradability characteristics.

## Results and Discussion

The preparation of PNMs was initiated by the self‐assembly of biodegradable poly(ethylene glycol)‐*b*‐poly(d,l‐lactide) (PEG‐PDLLA) block copolymers containing a pH‐cleavable linker (pH‐sensitive benzoic‐imine bond) to facilitate effective breakdown within the cell at acidic pH (pH 6.5).^21^ Photothermal features were introduced in the form of a hemispherical coating of gold, which undergoes plasmonic heating upon NIR laser irradiation. The resulting nanomotors were subsequently tested for their NIR‐mediated motility, and ability to facilitate disruption of biological membranes via the photothermal effect—enabling the self‐assembled constructs to pass through the cell membrane. Using this approach, we demonstrate the ability to deliver a ubiquitous anticancer drug, doxorubicin (Dox), as model drug, and fluorescein‐labelled bovine serum albumin (FITC‐BSA) as macromolecular cargo into the cells. Furthermore, we utilized such PNMs as functional carriers to activate enzymatic catalysis within living cells. This was achieved by the PNMs‐mediated cellular delivery of profluorescent substrate (fluorescein di‐β‐d‐galactopyranoside, FDG) alongside an appropriate enzyme (β‐galactosidase, β‐gal).

Biodegradable poly(ethylene glycol)‐*b*‐poly(d,l‐lactide) (PEG‐PDLLA) was utilized as the principal building block for PNMs, owing to its ease of self‐assembly and established biodegradability/compatibility (Scheme S1 in the Supporting Information). A pH‐sensitive benzoic‐imine bond was introduced as a linker between the two blocks to create polymeric chains of which the PEG chain would be easily cleaved at acidic pH, facilitating hydrolysis of the lactide block. Copolymer synthesis was performed similar to our previous work on PEG‐PDLLA polymersomes—and the final block copolymer structure was confirmed using gel permeation chromatography (GPC) and proton nuclear magnetic resonance (^1^H‐NMR) (Table S1 and Figure S1).^22^ Using the solvent‐switch methodology, self‐assembly of the PEG_44_‐PDLLA_115_ copolymer into polymersomes was accomplished.^22^ Spherical polymersomes with uniform size were observed using scanning electron microscopy (Figure S2). The pH‐sensitive behavior of the polymersomes was monitored by dynamic light scattering (DLS) and scanning electron microscopy (SEM; Figure S3). The results showed that the size of the polymersomes was slightly bigger at pH 6.5 due to the hydrolysis of the polymersomes, and consequent formation of aggregates—this was confirmed by SEM images (Figure S3B). Cumulative Dox release from polymersomes at different pH values (6.5 and 7.4) was measured (Figure S4). Faster release was observed at pH 6.5, when compared to the release at pH 7.4. This can be attributed to the existence of a pH‐cleavable linker that facilitates hydrolysis of polymersomes at acidic pH. The assembled polymersomes were further functionalized using a gold (Au) coating through a process of sputter coating. A significant color change of the polymersome solution was directly observed after the process (Figure S2C). The successful introduction of a hemispherical gold surface coating was evident from analysis using SEM (Figure 1 B and Figure S2D/E) and energy‐dispersive X‐ray spectroscopy elemental mapping analysis (Figure S5). By depositing a hemispherical gold coating, a Janus inorganic/organic hybrid construct that is capable of functioning as a photoactivatable nanomotor (PNM) was thus created. Our group has demonstrated the applicability of hybrid nanomotors for biomedical research.^23^ Indeed, the combination of organic and inorganic components, with their diverse functional attributes, in a single system is a route towards the development of hybrid materials with improved properties.

To investigate the autonomous motion of our nanomotors, PNMs were activated using a two photon‐confocal laser scanning microscope (TP‐CLSM) as a NIR laser source, and their movement behavior was recorded. Upon irradiation with NIR light, hybrid PNMs underwent autonomous motion, in a direction opposite to the source of light. The hypothesized mechanism of propulsion, which was previously shown to drive motility of Janus motors,^24^ is photo‐induced thermophoresis.^25^ The Au‐hemisphere undergoes plasmonic absorbance, resulting in an increase of the particle temperature and subsequent generation of a temperature gradient, which results in positive thermodiffusion (particles move from hot to cold regions).^26^

Due to their high temperature, particles acquire sufficient kinetic energy to drive their propulsion.^27^ Plotting the trajectories of PNMs and extracting the relative velocities as a function of laser power provided clear evidence for the successful photo‐activation of motility (Figure 1 C–E). There was no apparent lag time in the NIR‐activation of PNMs; particle motion switched from Brownian to directional concurrently upon laser irradiation (Figure S6). This was also confirmed by the (linear) shape of the extracted trajectories (Figure 1 E and Figure S7) and the analysis of their mean square displacement (MSD) curves (motion analysis is described in the Supporting Information), which displayed a shift from linear to non‐linear (parabolic) fitting (Figure 1 C). The PNMs displayed velocities ranging from 1.9±0.25 μm s^−1^ to 6.2±1.10 μm s^−1^, controlled by the laser power. In order to further demonstrate the NIR propelled motion of PNMs, nanoparticle tracking analysis (NTA) was used to track and observe their motion (Video S1 to Video S5). As negative controls, gold shells and polymersomes without a gold coat were investigated for their motion under NIR irradiation. The corresponding MSD and motion pathway are shown in Figure S8. From these results it is clear that only the Janus particle morphology leads to efficient enhanced diffusion.

**Figure 2:**
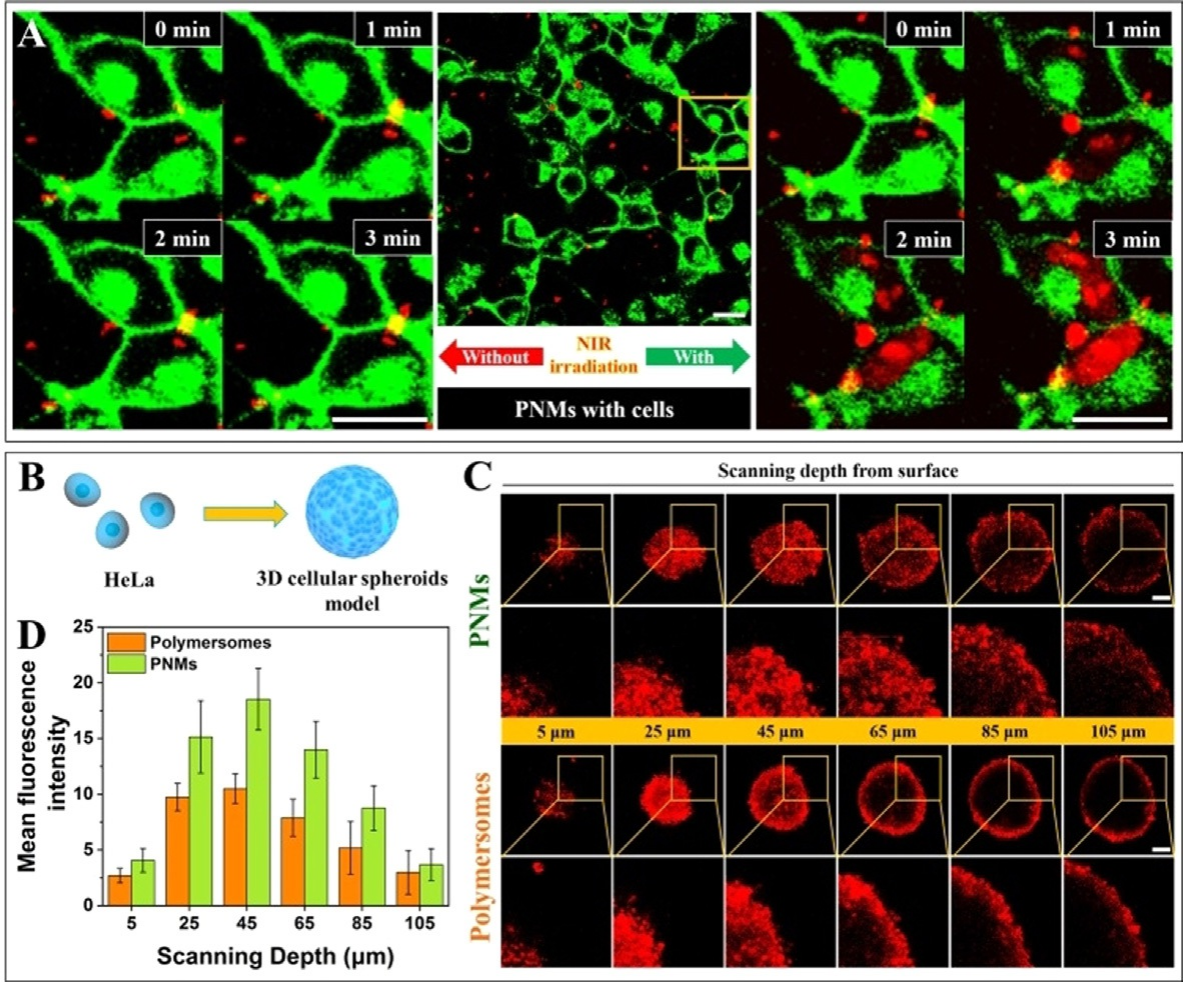
Intracellular delivery of small molecular cargoes and enhanced tissue penetration of PNMs via the assistance of NIR laser irradiation. A) Time‐lapsed CLSM images of 2D HeLa cells in presence of PNMs and PI without (left) and with (right) NIR laser irradiation. PNMs and PI were co‐added to the cell medium immediately before a standardized time‐scanning imaging sequence. The cell membrane was stained with Alexa Fluo^TM^ 488 conjugated wheat germ agglutinin. The red signals were obtained from Dox/PI. Scale bar=20 μm. B) Schematic illustration for the construction of a 3D HeLa tumor spheroid model. C) Z‐scanning CLSM images of 3D HeLa tumor spheroids to characterize the tumor penetration of PNMs and polymersomes (without Au coat) after 2 h culturing and NIR irradiation. The surface of the tumor spheroids was defined as 0 μm. Scale bar=100 μm. D) The mean fluorescence intensity (MFI) of 3D HeLa tumor spheroids.

Inspired by previous pioneering work,^28^ which showed the ability of light‐propelled micro/nanomotors to display enhanced membrane penetration, PNMs were envisioned to traverse robust biological barriers, which in turn can be utilized to drive active transportation of functional cargoes over the cellular membrane. To this end, delivery of molecular cargo into cancer cells (in this case HeLa) in 2D and 3D formats was investigated so that their performance under controlled in vitro conditions could be tested. First, the biocompatibility of PNMs was assessed using non‐cancer (NIH/3T3), as well as cancer cells (HeLa); their impact on viability was minimal across all conditions studied in this work (Figure S9). PNMs were loaded with a fluorescent chemotherapeutic agent (doxorubicin, Dox) to study the direct delivery of small molecular cargo, alongside freely dispersed propidium iodide (PI) to assess the consequence of membrane disruption using TP‐CLSM. HeLa cells were pre‐stained with Alexa Fluor^TM^ 488 conjugate wheat germ agglutinin to show the boundary of the cells. The red fluorescent signal originating from Dox (encapsulated within the polymersome lumen)/PI (as an external payload) was used to localize the position of the cargoes. The same cell sample was examined before and after irradiation with light (Figure 2 A). Under NIR irradiation, PNMs were able to cross the cellular boundary, and internalize into cells in a time dependent manner. Notably, PI as fluorescent agent was used as indicator to reflect the cell permeability during the whole intracellular delivery process. Upon NIR irradiation, PI was indeed able to enter the cells, without affecting significantly the cell integrity (Figure S10 left). Also, no PI uptake was observed when PNMs were not activated (Figure S10 right), or NIR was used in the absence of PNMs (Figure S11). These results provide sufficient evidence that intracellular delivery only occurred in the presence of PNMs with NIR‐irradiation. In order to observe the destination of PNMs after entering into the cells, the fluorescent probes LysoTracker and Hoechst 33342 were used to stain lysosomes and cell nuclei, respectively. As shown in Figure S12, PNMs were taken up by cells via traditional endocytosis and localized in lysosomes in the absence of NIR irradiation. Photothermal treatment was then initiated using 5 min NIR laser irradiation (Figure S13). Under these conditions, the PNMs also co‐localized to a large extent within the nucleus, demonstrating that direct translocation of the particles had taken place.

Having confirmed the ability of our PNMs to deliver cargo intracellularly, we undertook to study their ability to penetrate 3D tumor tissue models as a result of their motion.^29^ Previous research has demonstrated that active delivery carriers with motile properties could significantly improve tissue penetration, enhance delivery efficiency, and enable effective therapy.^30^ A light‐responsive nanomotor prepared by mesoporous‐macroporous silica and Pt was demonstrated to be able to penetrate into three dimensional (3D) spheroids deeply for combined cancer therapy.30f

Due to the spatial complexity and heterogeneity of 3D tumor spheroids, they represent excellent robust models for the investigation of the capacity of our PNMs in traversing diverse biological barriers.^31^ To this end, 3D tumor spheroids were developed by culturing of HeLa cells in a 3D platform (Figure 2 B), according to previously published protocols.^32^ Once the diameter of the cultured 3D HeLa spheroids reached approximately 500 μm, hydrophilic Dox‐loaded PNMs and polymersomes (without Au coat) were introduced to two separate tumor cultures. After 2 h, both cell cultures were extensively washed with PBS buffer, ensuring complete removal of the unattached polymersomes. Utilizing two photon‐confocal laser scanning microscopy, the capacity of both PNMs and polymersomes to penetrate tumor cells was monitored (Figure 2 C). In order to analyze the entirety of 3D‐HeLa spheroids, *z*‐scanning imaging sequences (*z*‐stacks) were conducted to observe the fluorescence intensity at different depths. The surface of the tumor spheroids was defined as 0 μm. Upon NIR irradiation, PNMs showed a uniform distribution and enhanced penetration. To quantify the measured fluorescence, CLSM images were processed and analyzed with ImageJ. The fluorescence intensity of PNMs was determined to be approximately 18.5 % at 45 μm, 14.0 % at 65 μm, and 8.8 % at 85 μm. Polymersomes, on the other hand, showed weaker fluorescent intensity of around 10.5 % at 45 μm, 7.9 % at 65 μm, and 5.2 % at 85 μm. PNMs, compared with bare polymersomes, displayed enhanced penetration upon NIR laser irradiation (Figure 2 D). Furthermore, experiments to evaluate the photothermal‐induced toxicity of NIR‐activated PNMs to cancer cells were conducted. To this end, PNMs were incubated with 3D HeLa spheroids for 2 h. Significant toxicity was observed upon NIR irradiation (Figure S14). Moreover, control experiments where 3D spheroids were either irradiated with NIR or incubated with inactivated PNMs did not show any toxicity.

For evaluation of the PNMs‐assisted intracellular delivery of macromolecules, FITC‐BSA was selected as macromolecular cargo. Cells (HeLa) were divided randomly into five groups to evaluate the effect of traversing the plasma membrane, including PNMs with and without NIR irradiation, polymersomes (without Au coat) with and without NIR irradiation, as well as cells exposed directly to the NIR laser. Free FITC‐BSA and PNMs/ polymersomes (without Au coat) were co‐introduced to the HeLa cells before performing the fluorescence imaging experiments by TP‐CLSM. BSA is a large protein (Mw=66 kDa) and thus, its intracellular uptake via the normal endocytosis process is rather challenging, as the cell membrane represents a stern natural barrier.^33^

**Figure 3:**
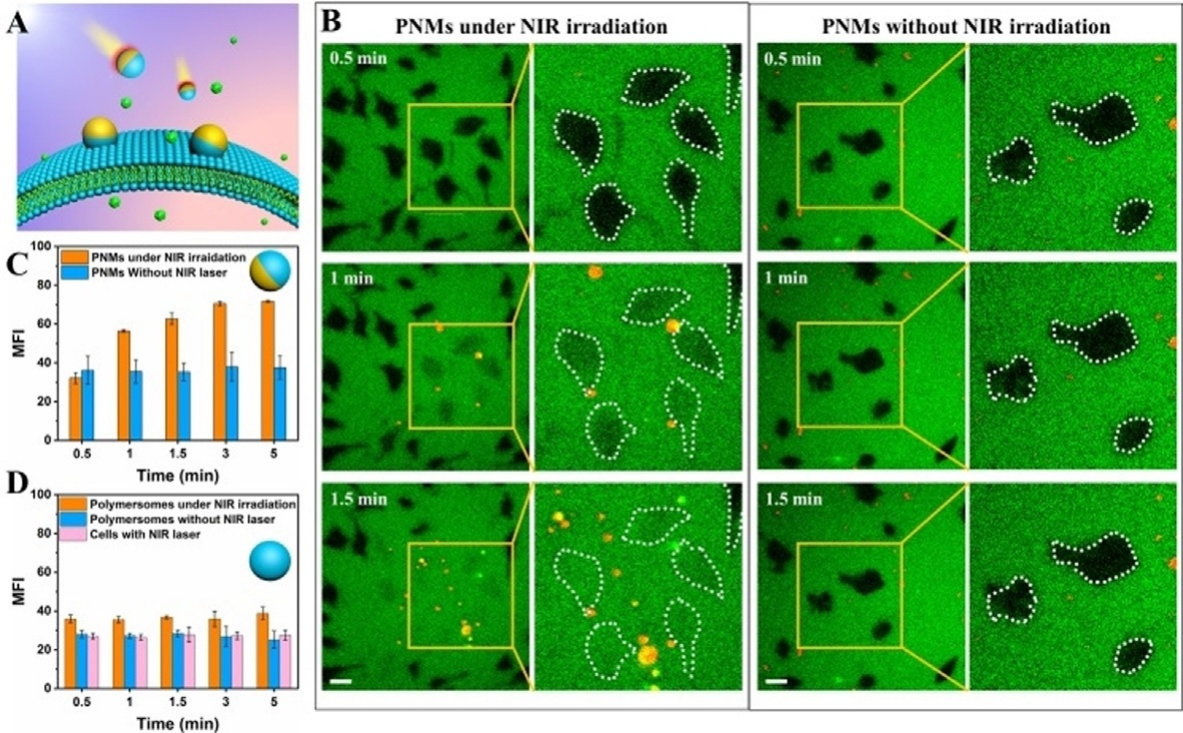
PNMs‐mediated intracellular delivery of macromolecules (FITC‐BSA) as characterized by TP‐CLSM. FITC‐BSA was introduced into the cell medium immediately before the TP‐CLSM measurement. A) Schematic illustration of PNM‐mediated intracellular delivery of FITC‐BSA upon NIR‐irradiation. B) Time‐lapsed TP‐CLSM images of HeLa cells in presence of PNMs with (left) and without (right) NIR laser irradiation. Green fluorescent signal comes from FITC‐BSA. Scale bar=20 μm. Mean fluorescence intensity (MFI) of HeLa cells as a function of time in presence of PNMs (C), and polymersomes (without Au coat) as well as HeLa cells directly exposed to NIR laser irradiation (D).

Indeed, before NIR irradiation, the uptake of FITC‐BSA was denied by the plasma membrane within each group (Figure 3). After NIR illumination and within 1 min, FITC‐BSA began to translocate intracellularly, as evident by the monitored fluorescence from confocal microscopy measurements (Presented in Video S6 to Video S10). After 5 min, fluorescence intensities increased by ca. 55 % of the irradiated cells in presence of PNMs (Figure 3 B, C and Figure S15). In comparison, control groups before and after NIR illumination did not show a change in intracellular fluorescence (Figure 3 D and Figure S16)—demonstrating the selective features of using PNMs for intracellular delivery of macromolecules.

**Figure 4:**
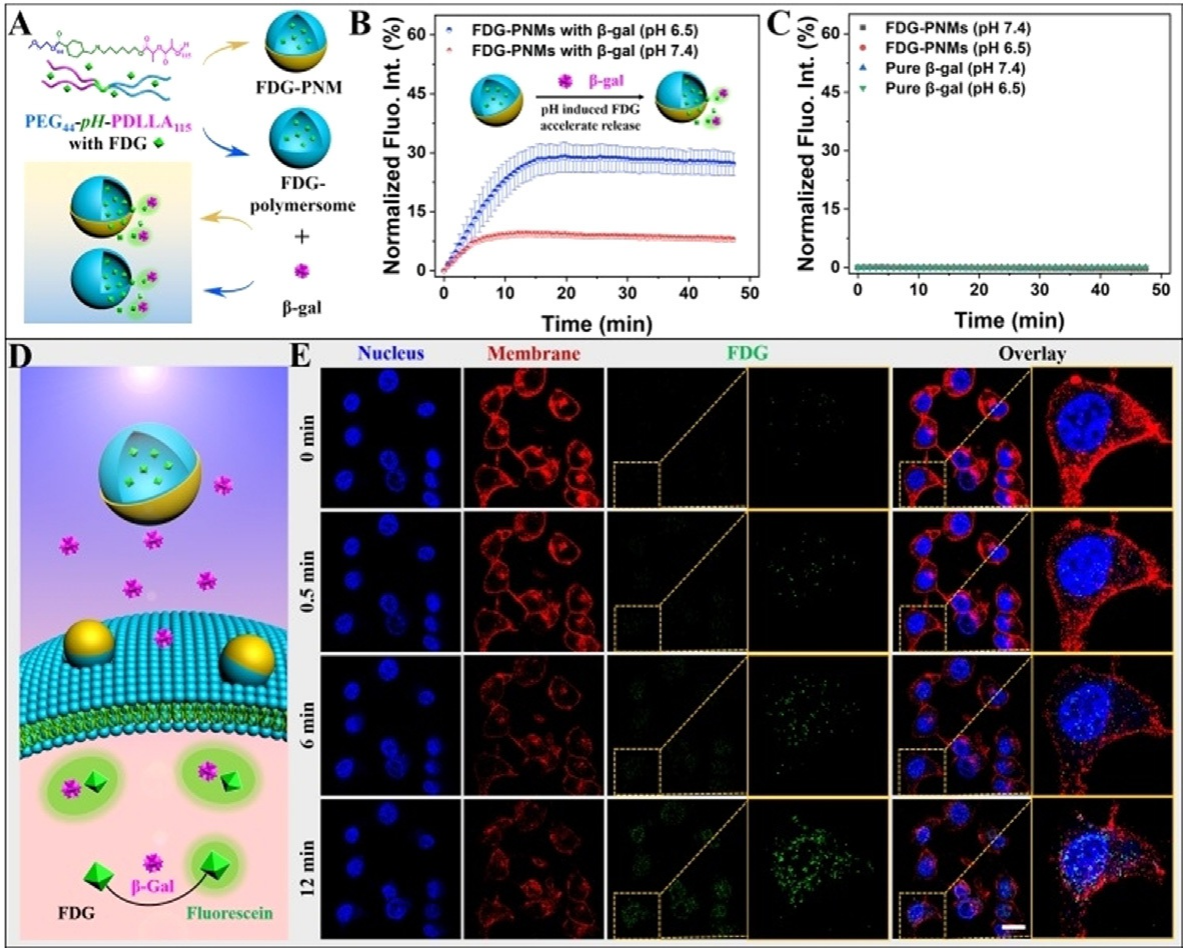
Co‐delivery of small molecules (enzyme substrate FDG) and macromolecules (enzyme β‐gal) via PNMs. A) Schematic illustration of release of non‐fluorescent FDG from polymersomes, and subsequent hydrolysis by β‐gal, resulting in the detection of fluorescent fluorescein. B) Kinetic study after the enzymatic activity at pH 6.5 and pH 7.4 as a function of time by measuring the increase in fluorescence in a microplate reader. C) Normalized fluorescence intensity in the control groups of FDG‐PNMs and pure β‐gal. D) Design strategy of nanomotor‐mediated transportation of both substrate (FDG, inside the motors) and enzyme (β‐gal, in solution), in living cells. E) Fluorescence increase in living cells before and after NIR irradiation characterized by TP‐CLSM. The permeabilization of the cell membrane under NIR irradiation mediated by the PNMs allows both the FDG‐PNMs and β‐gal to be transported into HeLa cells. Scale bar=20 μm.

Having confirmed the ability of our PNMs to facilitate intracellular delivery of small molecules (PI) and macromolecules (BSA), we further investigated whether such system can be utilized as multifunctional transporter—able to deliver enzyme substrate (molecular payload) and enzyme (macromolecular cargo) simultaneously, and undertake reactions within cells. To do this, profluorescent enzyme substrate (fluorescein di(β‐d‐galactopyranoside), FDG) was encapsulated in our PNMs (FDG‐PNMs) and polymersomes (FDG‐polymersomes), respectively. The enzyme β‐galactosidase (β‐gal) is able to convert the substrate FDG into fluorescein, which can be assayed by fluorescence spectroscopy (Figure 4 A).^34^

First experiments were conducted with free enzyme in PBS buffer of pH 6.5 and pH 7.4, respectively to which the FDG‐loaded polymersomes or PNMs were added. The kinetics of the reaction was monitored by a microplate reader through detecting the fluorescent product. As shown in Figure 4 B, an increase in fluorescence was both observed at pH 6.5 and pH 7.4, nevertheless the enzymatic rates were much higher in the group of pH 6.5, due to the acidic pH‐induced accelerated release of FDG. The negative control experiments all showed as expected no fluorescence development (Figure 4 C). Next it was investigated if the enzymatic reaction could be induced intracellularly by co‐delivery of FDG‐PNMs along with β‐gal to HeLa cells (Figure 4 D). FDG‐PNMs were readily introduced into HeLa cells when exposed to NIR illumination. Once the released FDG from PNMs was hydrolyzed by the co‐delivered β‐gal, non‐fluorescent FDG was converted to fluorescein intracellularly, lighting up the cells. Corresponding time‐lapsed images were made to track this enzymatic process by TP‐CLSM, as shown in Figure 4 E and Video S11. Both the cell membrane and cell nucleus were pre‐stained with Alexa Fluor^TM^ 594 conjugate wheat germ agglutinin and Hoechst 33342, respectively. Within 12 min, a fluorescent signal was observed intracellularly, which gradually increased over time. As a control, two separate experiments were performed with cells exposed to FDG‐PNMs/β‐gal in the absence of NIR irradiation (Figure S17), and cells directly exposed to the NIR laser (Figure S18). Indeed, no fluorescence was observed in both situations, demonstrating the necessity of NIR‐assistance for intracellular delivery, and highlighting the ability of our PNMs to transfer molecules/macromolecules to cells and allow reactions to take place.

## Conclusion

In summary, we have shown the design of a photothermally driven biodegradable nanomotor (PNM), which is based on hemispherical gold‐coated poly(ethylene glycol)‐benzoic‐imine‐poly(d,l‐lactide) (PEG‐PDLLA) polymersomes. Upon irradiation with near‐infrared light, these nanomotors undergo autonomous motion, with velocities ranging from 1.9±0.25 μm s^−1^ to 6.2±1.10 μm s^−1^, as a function of the laser power. Such nanomotors were able to traverse biological membranes and effectively penetrate tumor tissues, permitting active transport of molecular and macromolecular cargo either by encapsulation or co‐delivery—offering opportunities for their application in tissue penetration and cargo/drug delivery. Furthermore, the ability of PNMs to be loaded with functional components that can be released intracellularly can permit enzymatic catalysis to take place in living cells. The work provides a novel approach to designing active nanomaterials for future application in the biomedical field. Research to explore the utility of such PNMs in the active transportation of assorted macromolecular cargoes, as well as their interaction with micro‐sized motors is in progress.

## Conflict of interest

The authors declare no conflict of interest.

## Supporting information

As a service to our authors and readers, this journal provides supporting information supplied by the authors. Such materials are peer reviewed and may be re‐organized for online delivery, but are not copy‐edited or typeset. Technical support issues arising from supporting information (other than missing files) should be addressed to the authors.

Supplementary

Supplementary

Supplementary

Supplementary

Supplementary

Supplementary

Supplementary

Supplementary

Supplementary

Supplementary

Supplementary

Supplementary
