# Supplementary material for: Photoactivated Polymersome Nanomotors: Traversing Biological Barriers
Source: Angew Chem Int Ed Engl. 2020 Jul 27;59(39):16918–25. doi: 10.1002/anie.202003748 (PMC7540338; doi:10.1002/anie.202003748)
Supplement: Supplementary file 1 — Supplementary [file ANIE-59-16918-s001.pdf]

Angewandte Chemie
Eine Zeitschrift der Gesellschaft Deutscher Chemiker

Supporting Information

Photoactivated Polymersome Nanomotors: Traversing Biological Barriers

Jingxin Shao+, Shoupeng Cao+, David S. Williams, Loai K. E. A. Abdelmohsen, and Jan C. M. van Hest**

anie_202003748_sm_miscellaneous_information.pdf
anie_202003748_sm_Video_S1.wmv
anie_202003748_sm_Video_S10.wmv
anie_202003748_sm_Video_S11.wmv
anie_202003748_sm_Video_S2.wmv
anie_202003748_sm_Video_S3.wmv
anie_202003748_sm_Video_S4.wmv
anie_202003748_sm_Video_S5.wmv
anie_202003748_sm_Video_S6.wmv
anie_202003748_sm_Video_S7.wmv
anie_202003748_sm_Video_S8.wmv
anie_202003748_sm_Video_S9.wmv

Supporting Information
©Wiley-VCH 2020
69451 Weinheim, Germany

Abstract: Synthetic nanomotors are appealing delivery vehicles for the dynamic transport of functional cargo. Although there are literature examples of such systems, their effective translation toward biological applications is limited due to the use of non-degradable components. Furthermore, the limitation of size has been an impediment to application due to the importance of achieving nanoscale (ca. 100 nm) dimensions, as opposed to microscale examples that are far more prevalent in the literature. Here, we present a hybrid nanomotor that can be activated by near-infrared (NIR)-irradiation for the triggered delivery of internal cargo and facilitated transport of external agents to the cell. Utilizing biodegradable poly(ethylene glycol)-*b*-poly(D,L-lactide) (PEG-PDLLA) block copolymers, with the two blocks connected via a pH sensitive imine bond, we generate nanoscopic polymersomes that are subsequently modified with a hemispherical gold nanocoat. As a consequence of the Janus morphology, such hybrid polymersomes are capable of undergoing photothermal motility in response to thermal gradients generated by plasmonic absorbance of NIR irradiation, with velocities ranging from 1.9 ± 0.25 μm s^{-1} to 6.2 ± 1.10 μm s^{-1} in response to modulating laser power. These biodegradable photothermally-driven polymersome nanomotors (PNMs) are capable of traversing cellular membranes, allowing the intracellular delivery of molecular and macromolecular cargo, and as such hold great potential for biomedical applications.

DOI: 10.1002/anie.2020XXXXX

Table of Contents

1. Materials
2. Instruments
2.1 Nuclear Magnetic Resonance Spectroscopy (NMR)
2.2 Gel Permeation Chromatography (GPC)
2.3 Dynamic Light Scattering (DLS)
2.4 Scanning Electron Microscopy (SEM)
2.5 Two photon-Confocal Laser Scanning Microscopy (TP-CLSM)
2.6 Microplate Reader
2.7 pH meter
2.8 UV-vis spectroscopy
2.9 NanoSight
3 Synthesis and Methods
3.1 Synthesis of poly(ethylene glycol)-benzoic-imine-poly(D,L-lactide) (PEG-PDLLA)
3.2 Preparation and characterization of polymersomes and hemispherical gold-coated polymersome nanomotors (PNMs)
3.3 pH-sensitive behaviors
3.4 Doxorubicin (Dox) release as a function of pH value
3.5 Near-infrared (NIR)-activated motility of PNMs
3.6 Cell culture
3.7 Cytotoxicity evaluation by MTT assay
3.8 PNMs-intracellular delivery of encapsulated Dox
3.9 Intracellular localization of PNMs
3.10 3D cellular spheroids penetration
3.11 Therapeutic evaluation towards 3D cellular spheroids
3.12 PNM-mediated intracellular delivery of fluorescein isothiocyanate conjugate albumin from bovine serum (FITC-BSA)
3.13 Preparation of fluorescein di-β-D-galactopyranoside (FDG) loaded PNMs (FDG-PNMs) and its intracellular delivery alongside β-Galactosidase (β-Gal)
4. Supplementary figures and tables
5. References
6. Supplementary videos
Video S1. Nanoparticle tracking video of PNMs in the absence of NIR laser irradiation
Video S2. Nanoparticle tracking video of PNMs under NIR laser irradiation
Video S3. Nanoparticle tracking video of gold nanoshells without NIR laser irradiation
Video S4. Nanoparticle tracking video of gold nanoshells under NIR irradiation
Video S5. Motion behavior of polymersomes (without gold coat) under NIR irradiation
Video S6. Intracellular delivery of FITC-BSA via PNMs under NIR irradiation
Video S7. Co-introduction of polymersomes and FITC-BSA to HeLa cells under NIR irradiation
Video S8. Irradiation of HeLa cells with NIR laser
Video S9. Intracellular delivery of FITC-BSA via PNMs without NIR irradiation
Video S10. Co-introduction of polymersomes and FITC-BSA to HeLa cells without NIR irradiation
Video S11. Co-delivery of enzyme substrates (FDG) and enzyme (β-gal) via PNMs under NIR irradiation

Experimental Procedures

1. Materials

All chemicals were used as received unless otherwise stated. Poly(ethylene glycol) methyl ether (mPEG, M_n 2 kDa) was purchased from Rapp Polymers. 4-formylbenzoic acid (97%), N-(3-dimethylaminopropyl)-N-ethylcarbodiimide hydrochloride (EDC) (98%), 4-(dimethylamino)pyridine (DMAP) (>99%), 6-amino-1-hexanol (97%), 1,8-diazabicyclo[5.4.0]undec-7-ene (DBU) (98%) and β-galactosidase from *Escherichia coli* (β-gal) were obtained from Sigma-Aldrich. 1,4-Dioxane and tetrahydrofuran (THF) were obtained from Biosolve Chimie. Dialysis Membrane MWCO 12,000 -14,000 Da from Spectra/Pro® was used for dialysis. Sodium chloride and ultrafree-CL Centrifugal Filter (0.45 μm) were obtained from Merck. Fluorescein isothiocyanate conjugate albumin from bovine serum (FITC-BSA), Wheat Germ Agglutinin, Alexa Fluor™ 488 Conjugate, Wheat Germ Agglutinin, Alexa Fluor™ 594 Conjugate, Hoechst 33342, LysoTracker™ Green DND-26, Calcein-AM, propidium iodide (PI), Dulbecco's modified eagle medium (DMEM), phosphate-buffered salines (PBS, pH 7.4), no mycoplasma fetal bovine serum (FBS), trypsin-EDTA, and penicillin-streptomycin were obtained from ThermoFisher. Gold nanoshells were purchased from nanoComposix. Doxorubicin hydrochloride (Dox · HCl) was purchased from Fluorochem. Fluorescein di(β-D-galactopyranoside) (FDG) was purchased from Carbosynth. Ultrapure Milli Q (Millipore) water (18.2 MΩ·cm) was used in this work.

2. Instruments

2.1 Nuclear Magnetic Resonance Spectroscopy (NMR): Routine proton nuclear magnetic resonance (^{1}H-NMR) measurements were conducted on a Bruker Avance 400 MHz Ultrashield™ spectrometer equipped with a Bruker SampleCase auto-sampler, using $CDCl_3$ as the solvent and TMS as the internal standard.
2.2 Gel Permeation Chromatography (GPC): The molecular weights and dispersity index of the copolymers were characterized by using a Prominence-I GPC system (Shimadzu) with a PL gel 5 μm mixed D (Polymer Laboratories) and equipped with a RID-20A differential refractive index detector. Polystyrene standards were used for calibration. THF was used as an eluent, with a flow rate of 1 mL per minute.
2.3 Dynamic Light Scattering (DLS): The hydrodynamic size of the polymersomes was measured using a Malvern Instruments Zetasizer (model Nano ZSP) equipped with a 633 nm He-Ne laser and an avalanche photodiode detector. Zetasizer software was used to process and analyze the data.
2.4 Scanning Electron Microscopy (SEM): FEI Quanta 200 3D FEG was used to characterize the morphology of the polymersomes and Janus structures.
2.5 Two photon-Confocal Laser Scanning Microscopy (TP-CLSM): Fluorescent images were observed and recorded using CLSM (Zeiss LSM510 META NLO, and Leica TCS SP5X) equipped with two-photon laser source (Chameleon Vision, Coherent, USA).
2.6 Microplate Reader: Cell viability was evaluated via a microplate reader (Safire^2, TECAN). The reaction progress was monitored via the fluorescent signal from fluorescein on the Spark ® 10M microplate reader (TECAN).
2.7 pH meter: FiveEasy Plus™ FEP20 pH Meter (METTLER TOLEDO) was used to monitor the pH.
2.8 UV-vis spectroscopy: Cumulative Dox release at 37 °C in PBS buffer was characterized using UV-vis spectroscopy (V-650, JASCO).
2.9 NanoSight: NanoSight LM10HS instrument (Malvern Instruments) equipped with an Electron Multiplication Charge Coupled Device camera and external laser source (660 nm, BeamQ Lasers) was utilized to track the motion of PNMs, polymersomes, and gold shells.

3. Synthesis and Methods

3.1 Synthesis of poly(ethylene glycol)-benzoic-imine-poly(D,L-lactide) (PEG-PDLLA)

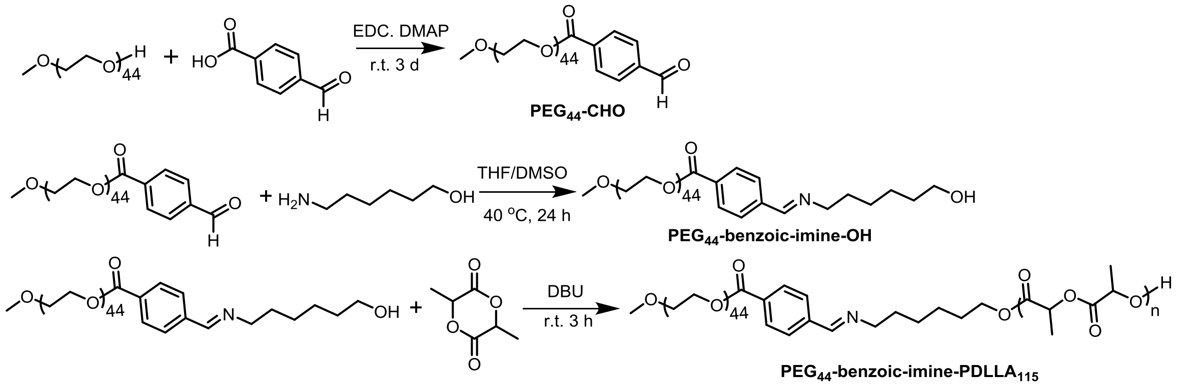

Scheme S1. Scheme outlining the synthesis of poly(ethylene glycol)-benzoic-imine-poly(D,L-lactide) (PEG_{44}-$PDLLA_{115}$) copolymer.

Synthesis of PEG_{44}-CHO: The synthesis of aldehyde modified PEG was performed according to a previously reported literature procedure.[1] Firstly, PEG (4 g, 2 mmol), 4-carboxybenzaldehyde (1.5 g, 10 mmol), EDC·HCl (3.85 g, 20 mmol) and DMAP (0.06 g, 0.5 mmol) were dissolved in 100 mL dichloromethane (DCM) and stirred for 48 h at 25 °C. After the reaction was completed, the solution was concentrated by rotary evaporation, and then the mixture was washed with saturated NaCl solution (3 times). Subsequently, the organic layer was collected and dried with anhydrous magnesium sulfate. After filtration, the filtrate was concentrated and precipitated twice in excess cold diethyl ether. The final product was dried under vacuum at room temperature overnight and obtained as a white solid with a yield of 56%. ^{1}H-NMR (400 MHz, Chloroform-*d*) δ 10.11 (s, 1H), 8.23 – 8.21 (d, 2H), 7.97 – 7.95 (d, 2H), 4.53 – 4.50 (d, 2H), 3.64 (s, 174H), 3.38 (s, 3H). GPC (RI) for PEG_{44}-CHO: M_n (Đ) = 2.53 kDa (1.04).

Synthesis of PEG_{44}-benzoic-imine-OH: The synthesis of pH responsive PEG was performed according to a previously reported literature procedure.[2] 6-aminohexan-1-ol (1.17 g, 10 mmol) was added to a solution of PEG_{44}-CHO (2 g, 1 mmol)) in a mixture of tetrahydrofuran and DMSO (8/1, 9 ml), and the mixture was stirred at 40 °C for 12 h. The solvent was evaporated under reduced pressure. The product was precipitated in anhydrous cold ethyl ether twice to remove impurities and dried under vacuum; it was obtained as a white solid with a 89% yield. ^{1}H-NMR (400 MHz, Chloroform-*d*) δ 8.32 (s, 1H), 8.10– 8.08 (d, 2H), 7.80 – 7.79 (d, 2H), 4.50 – 4.48 (m, 2H), 3.64 (s, 176H), 3.38 (s, 3H). GPC (RI) for PEG_{44}-benzoic-imine-OH: M_n (Đ) = 2.66 kDa (1.04).

Synthesis of PEG_{44}-benzoic-imine-$PDLLA_{115}$ (PEG_{44}-$PDLLA_{115}$): The synthesis of pH responsive PEG-PDLLA was performed according to a previously reported literature procedure.[3] PEG_{44}-benzoic-imine-OH macro-initiator (0.1 mmol) was weighed into a round bottom flask along with D,L-Lactide (12 mmol). Then, dry toluene was added to the flask and the solvent evaporated in order to dry the contents before polymerization. The dried reagents were then re-dissolved in dry DCM (25 mL, [monomer] = 0.5M) and DBU was added (0.5 equiv. with respect to [initiator]; 0.1 mmol = 8 µL) under argon. The reaction was stored at room temperature (RT) for around 3 hours, until there was no evidence of the monomer from the ^{1}H-NMR spectra. After completion was confirmed by ^{1}H-NMR, the reaction mixture was quenched with excess benzoic acid and then precipitated into ice cold diethyl ether (100 mL) twice and the remaining wax was partially dried under nitrogen before dissolving in dioxane and lyophilisation to yield a white powder with a yield of 73%. ^{1}H NMR (400 MHz, Chloroform-*d*) δ 8.32 (s, 1H), 8.10 – 8.08 (d, 2H), 7.80 – 7.79 (d, 2H), 5.17 (m, 229H), 4.49 (t, J = 5.0 Hz, 2H), 3.64 (s, 174H), 3.38 (s, 3H), 1.62 – 1.51 (m, 696H). GPC (RI) for PEG_{44}-benzoic-imine-$PDLLA_{115}$: M_n (Đ) = 13.1 kDa (1.18).

3.2 Preparation and characterization of polymersomes and hemispherical gold-coated polymersome nanomotors (PNMs)

In a 15 mL vial PEG_{44}-$PDLLA_{115}$ (20 mg) was dissolved in 2 mL of a mixture of THF/dioxane (1:4, v/v). Thereafter, a magnetic stirring bar was added, and the vial was sealed with a rubber septum. Subsequently, 2 mL of ultrapure MilliQ water was added *via* a syringe pump (Chemyx Inc. Fusion 100 Syringe Pump) with a flow rate 1 mL h^{-1}. Afterwards, the obtained cloudy solution was transferred into a prehydrated dialysis bag (12-14 kDa, 2 mL cm^{-1}) and dialyzed against a pre-cooled NaCl solution (50 mM) at 5 ^oC for at least 24 h with a dialysis solution change after 1 h. For preparation of Janus polymersomes, the sputter coating technique was used following a previously published protocol.[4] Briefly, a droplet of polymersome solution was drop-cast onto a clean silicon wafer, followed by sputter coating using a turbo sputter coater (Quorum Technologies, K575X). Thereafter, Janus polymersomes were re-dispersed into Milli-Q water through ultrasound treatment. The morphology of polymersomes, before and after coating, was characterized using SEM and DLS. For Dox loading, 1 mg Dox was dissolved together with the block copolymer (20 mg). Similarly, FDG loading was performed by dissolving it (1mg) together with the block copolymer.

3.3 pH-sensitive behaviors

PBS with two pH values (pH 6.5 and pH 7.4) was prepared by tuning with 0.1 M HCl. Size changes of polymersomes in the presence of PBS buffer were monitored by DLS for 10 hours. Morphological changes of polymersomes as a function of pH value was observed by SEM.

3.4 Doxorubicin (Dox) release as a function of pH value

The release profile of Dox towards PBS buffer (pH 6.5 and pH 7.4) at 37 ^oC was calculated by measuring the absorbance intensity of released Dox in buffer at each time point using UV-vis spectroscopy.

3.5 Near-infrared (NIR)-activated motility of PNMs

The autonomous motion of PNMs was observed and recorded by TP-CLSM (Zeiss LSM510 META NLO) equipped with a ×63 oil immersion microscope objective. PNMs were detected by the fluorescent signal of the encapsulated Dox. Movement trajectories were tracked and analyzed by using ImageJ and Origin softwares. Based on the extracted trajectories, the velocity of NIR propelled PNMs (V) was determined by measuring both the travelled distance (D) and duration time (t) – following the formula: V = D/t. Corresponding mean square displacements (MSD) were then calculated following the reported equation: $MSD= (x(\Delta t)-x(0))^2+ (y(\Delta t)-y(0))^2$.[5] Nanoparticle tracking analysis (NTA) was also used to analyze the motion behavior of PNMs, polymersomes, and gold shells by using NanoSight. Samples were suspended in Milli-Q water to yield an approximate concentration of 10^7 and 10^8 particles per mL. 1 mL of samples was loaded in the NTA chamber via a syringe. Then, the motion of PNMs, polymersomes, and gold shells was recorded for 30 s. A 660 nm DPSS Red Diode Laser Max Output Power was utilized as external laser source to propel the particles. Two laser intensities were used during the whole experiment, namely 0 W (laser off) and 1W (laser on). The concentration of PNMs was 5.19×10^7 particles mL^{-1} (0 W) and 1.41×10^8 particles mL^{-1} (1 W), respectively. For gold nanoshells, 2.89×10^8 particles mL^{-1} (0 W) and 5.11×10^8 particles mL^{-1} (1 W). For polymersomes, the concentration was 3.31×10^8 particles mL^{-1} (0 W) and 3.89×10^8 particles mL^{-1} (1 W). The NTA 2.2 software allows the extraction and analysis of the trajectories of single particles. For each group, 30 nanoparticles were tracked for 30 seconds and MSDs and velocity were calculated according to the reported method.[6]

3.6 Cell culture

Human cervical cancer cells (HeLa) and mice embryonic fibroblast cells (NIR/3T3) were cultured in DMEM cell culture medium supplemented with 10% FBS and 1% penicillin-streptomycin in the cell incubator (ThermoFisher Scientific) at 37 ^oC with an atmosphere of 5% CO_2 and 70% humidity.

3.7 Cytotoxicity evaluation by MTT assay

Cytotoxicity evaluation of PNMs was conducted by using a standard 3-(4,5-dimethylthiazol-2-yl)-2,5-diphenyltetrazolium bromide (MTT) assay using a microplate reader (Safire^2, TECAN).

3.8 PNMs-intracellular delivery of encapsulated doxorubicin (Dox)

HeLa cells were seeded in µ-Slide 8 wells (ibidi) and incubated in DMEM cell culture medium. The cell membrane was stained with Wheat Germ Agglutinin, Alexa Fluor™ 488 conjugate to show the boundary of the cells. PNMs / polymersomes and PI were added before TP-CLSM characterization (Zeiss LSM510 META NLO).

3.9 Intracellular localization of PNMs

Pre-seeded HeLa cells in µ-Slide 8 wells were stained with Hoechst 33342 and LysoTracker™ Green DND-26 for the observation of the cell nucleus and lysosomes, respectively. After co-culture with PNMs (50 µL well, 2 mg mL^{-1}) for 4 h, HeLa cells were characterized by TP-CLSM (Leica TCS SP5X). The cells were washed thoroughly with PBS buffer for three times to remove free PNMs.

3.10 3D cellular spheroids penetration

3D spheroids better resemble the complex in vivo environment of cells, as compared to traditional 2D approaches, more realistically recapitulating the tumor microenvironment. As valuable tool for nanomedicine, 3D spheroid models could be used for (a) a more accurate drug screening, (b) a better understanding of molecular and cellular mechanisms, (c) the evaluation of potential therapeutic agents before further pre-clinical studies, (d) cell matrix interactions.[7] 3D HeLa cellular spheroids were prepared by seeding HeLa cells in agarose-coated 96-well plates, according to previously published protocols.[8] Briefly, 0.15 g agarose was added to 10 mL of low glucose DMEM (1.5% wt/vol) in an appropriate beaker, sealed with a lid and autoclaved at 120 ^{o}C for 20 min. Thereafter, the hot solution (~80 ^{o}C) was added to a 96-well plate (flat bottomed, 50 µL per well) under sterile conditions. The HeLa cell suspension in high glucose DMEM medium was seeded and cultured for the formation of 3D HeLa cellular spheroids. Before TP-CLSM (Zeiss LSM510 META NLO) measurement, 50 µL PNMs / polymersomes (2 mg mL^{-1}) were introduced to the 3D spheroids and co-cultured for 2 h, followed by careful washing with pre-warmed PBS buffer to remove all the free polymersomes (without Au coat) and PNMs.

3.11 Therapeutic evaluation towards 3D cellular spheroids

The therapeutic effect of PNMs was evaluated using 3D HeLa cellular spheroids. 3D HeLa spheroids were prepared by seeding HeLa cells in agarose pre-coated 96-wells plates. When the diameter of HeLa spheroids reached ca. 500 µm, the spheroids were moved to ibidi-8 wells and divided into two groups, including PNMs with/without NIR laser irradiation, and spheroids with/without NIR laser irradiation. Before characterization by TP-CLSM, 50 µL PNMs (2 mg mL^{-1}) were added to the 3D HeLa spheroids and co-cultured for 2 h. Calcein-AM and PI were then used to co-stain the spheroids for assessing the cell viability after photothermal treatment by TP-CLSM.

3.12 PNM-mediated intracellular delivery of fluorescein isothiocyanate conjugate albumin from bovine serum (FITC-BSA)

To evaluate the PNM-mediated active intracellular delivery of macromolecules, pre-cultured cells (in µ-slide 8 wells) were randomly divided into five groups, which included PNMs with/without NIR irradiation, polymersomes (without Au coat) with/without NIR irradiation, and cells only. For both PNM and polymersome groups, 50 µL of a 2 mg mL^{-1} particle dispersion was added separately to each well before NIR irradiation. 50 µL FITC-BSA (10 µg µL^{-1}) was then added to all the samples. TP-CLSM (Zeiss LSM510 META NLO) was used to observe and record the active intracellular transportation of FITC-BSA.

3.13 Preparation of fluorescein di-β-D-galactopyranoside (FDG) loaded PNMs (FDG-PNMs) and its intracellular delivery alongside β-Galactosidase (β-Gal)

FDG was encapsulated in the PNMs following the above described procedure (supporting information – section 3.2). Fluorescence (as a result of fluorescein production) was monitored using a microplate reader (Spark ® 10M, TECAN). For the reactions carried out in living cells, HeLa cells were pre-seeded and incubated in µ-slide 8 wells. FDG-PNMs (50 µL) and β-gal (20 µL) were co-introduced. Fluorescence increase was monitored and recorded using a TP-CLSM (Leica TCS SP5X). Wheat Germ Agglutinin, Alexa Fluor™ 594 conjugate, and Hoechst 33342 were used to stain the cell membrane and the nucleus, respectively.

4. Supplementary figures and tables

Table S1. GPC analysis of precursor polymer and amphiphilic block copolymer (PEG_{44}-$PDLLA_{115}$).

Polymer composition	M_n / kDa	GPC
		Đ
PEG_{44}-OH	2.02	1.04
PEG_{44}-CHO	2.53	1.04
PEG_{44}-benzoic-imine-OH	2.66	1.04
**PEG_{44}-benzoic-imine-$PDLLA_{115}$**	13.1	1.18

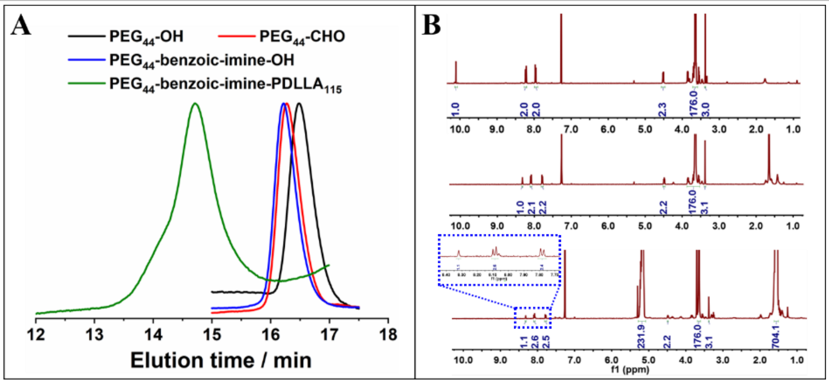

Figure S1. Characterization of PEG_{44}-$PDLLA_{115}$ amphiphilic block copolymer. (A) GPC traces. (B) ^{1}H-NMR spectra of PEG_{44}-OH, PEG_{44}-benzoic-imine-OH, PEG_{44}-benzoic-imine-$PDLLA_{115}$.

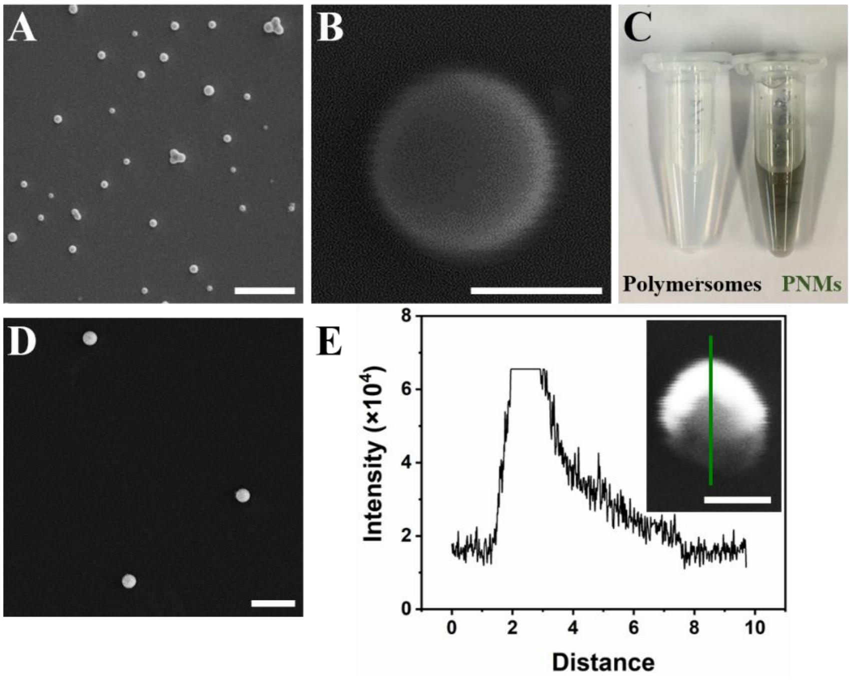

Figure S2. (A, B) SEM image of polymersomes before sputter coating. Scale bar = 1 μm and 100 nm, respectively. (C) Optical image of polymersomes before and after sputter coating. (D) SEM image of PNMs after sputter coating. Scale bar = 2 μm. (E) Intensity profile of the PNM (insert image, scale bar = 100 nm).

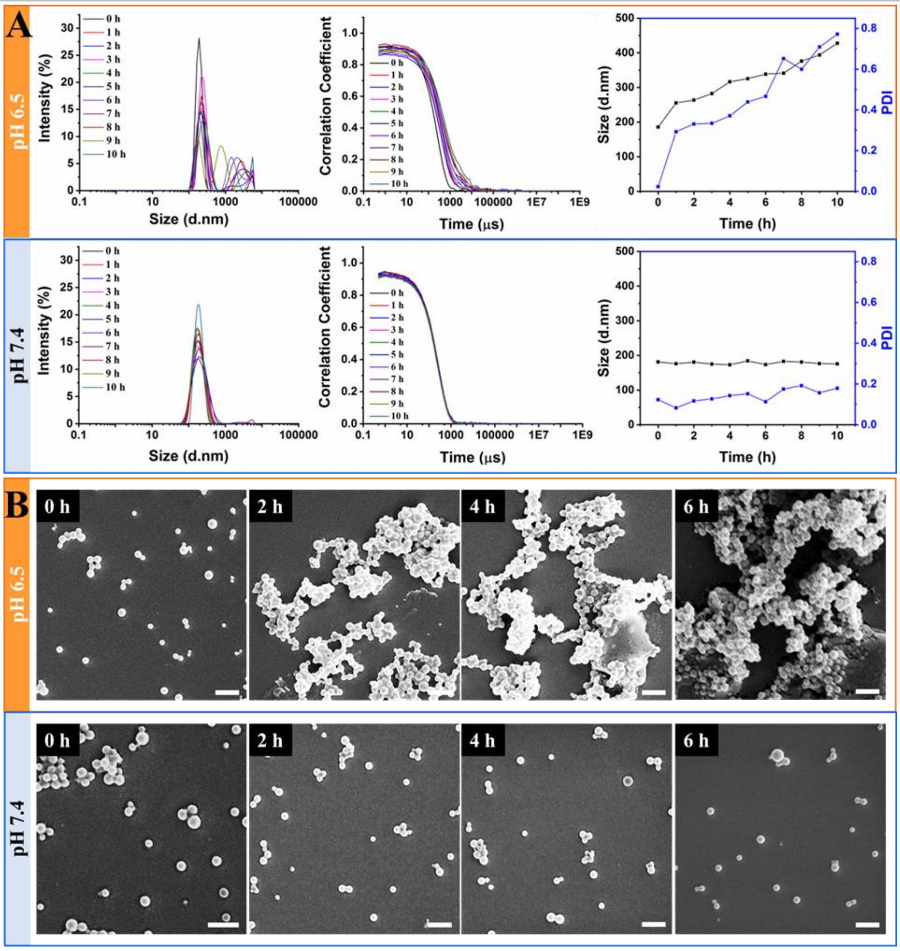

Figure S3. pH-responsive behavior of polymersomes. (A) Effect of pH values on the size of polymersomes in the presence of PBS (pH 6.5 / pH 7.4) measured by DLS for 10 h. (B) SEM images of morphological changes of polymersomes as a function of pH, scale bar = 500 nm.

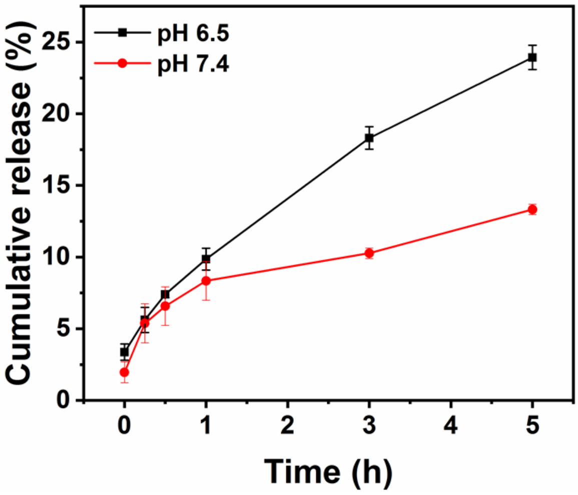

Figure S4. pH dependent cumulative release of Dox from polymersomes in PBS buffer (pH 6.5 / pH 7.4) at 37 °C.

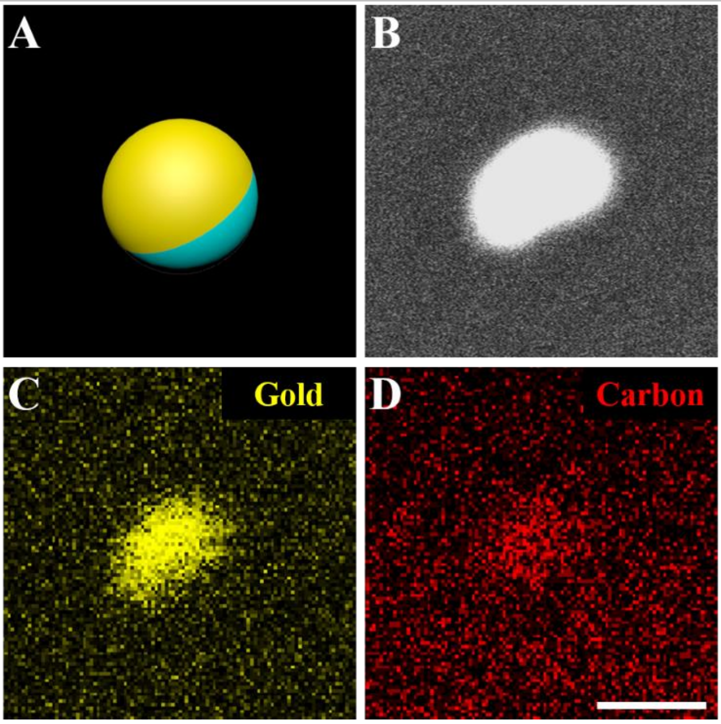

Figure S5. Energy-dispersive X-ray spectroscopy (EDX) elemental mapping analysis for PNMs, scale bar = 200 nm.

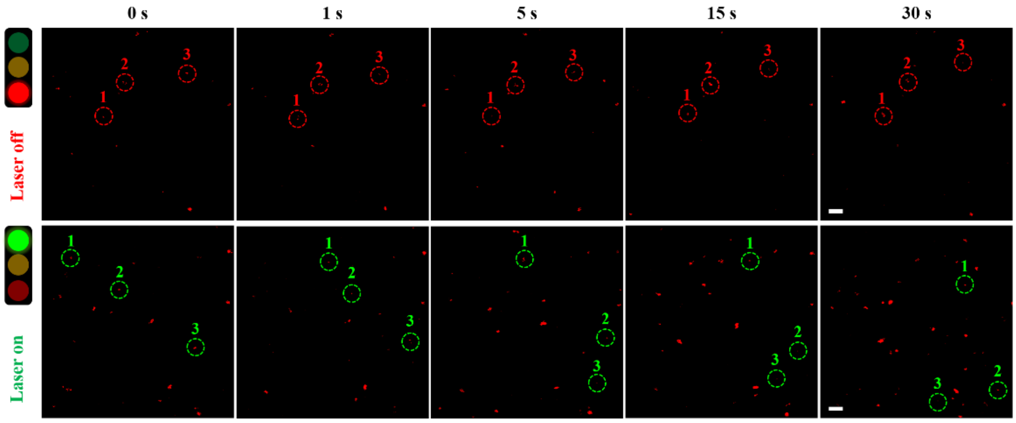

Figure S6. Time-lapse images of PNMs with and without NIR laser irradiation, scale bar = 10 μm.

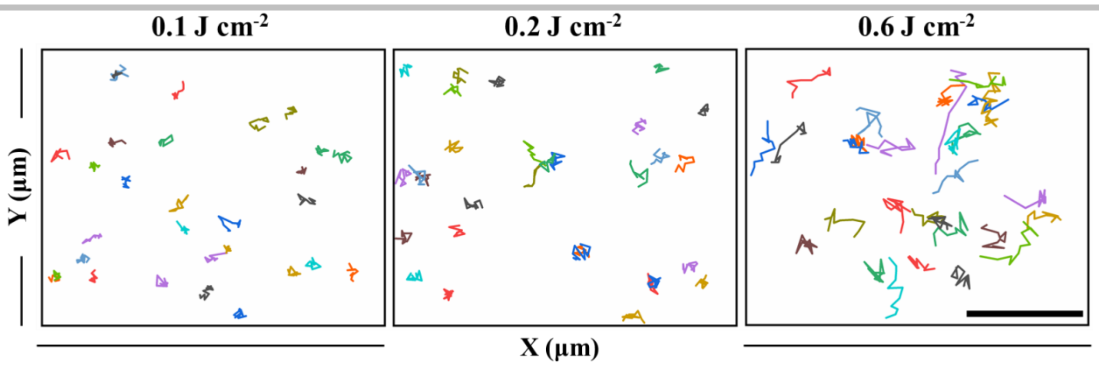

Figure S7. Movement trajectory of PNMs under NIR irradiation with different intensities (scale bar = 50 μm).

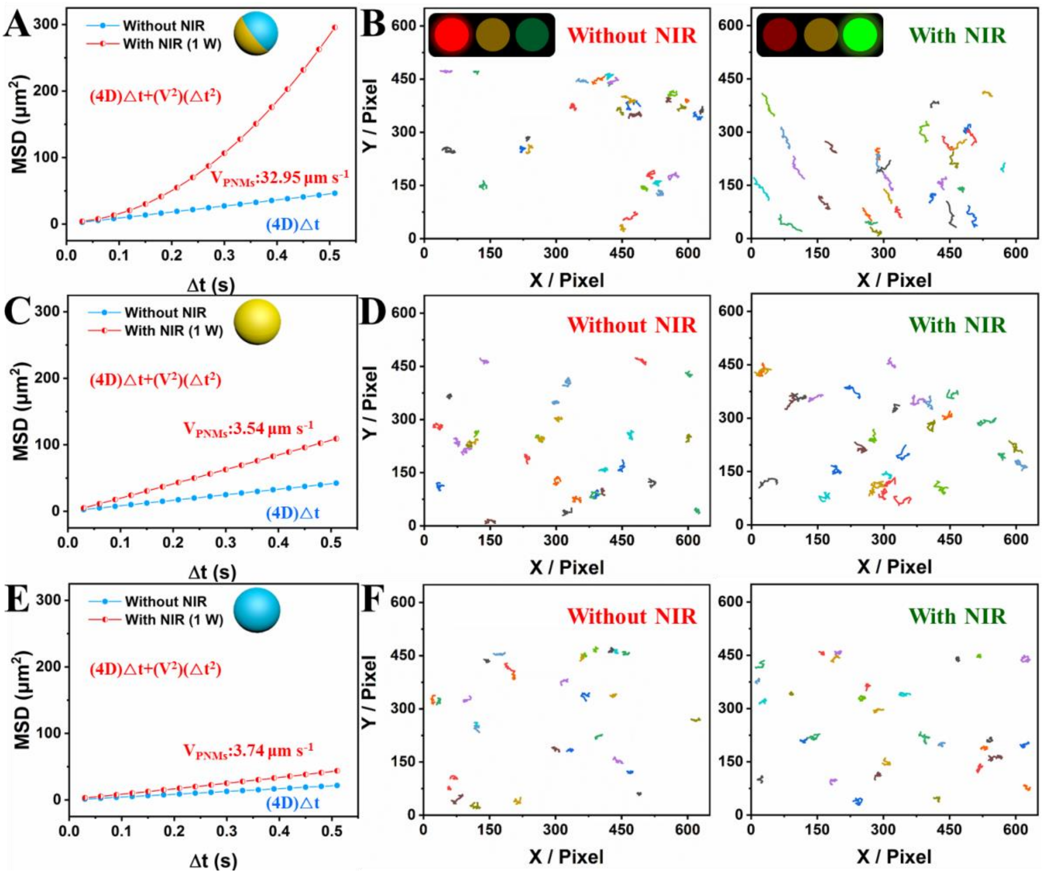

Figure S8. MSD and motion trajectory of PNMs (A, B), gold shells (C, D), and polymersomes without gold coating (E, F), which were obtained by nanoparticle tracking analysis (NTA).

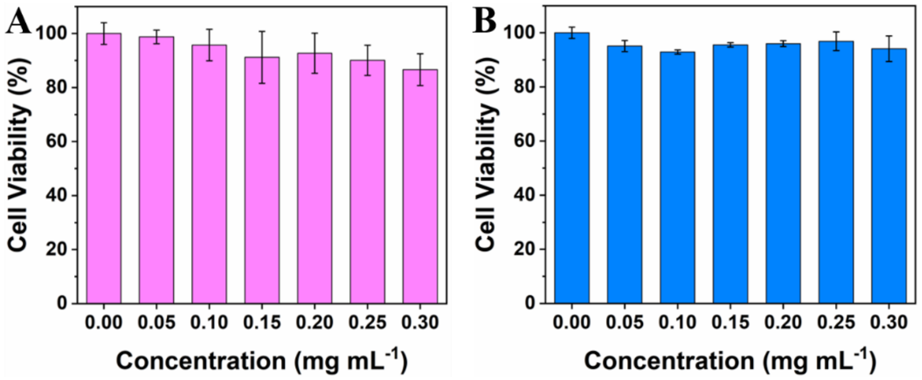

Figure S9. Cytotoxicity evaluation of PNMs as determined by using a standard MTT assay. (A) NIH/3T3 cells. (B) HeLa cells.

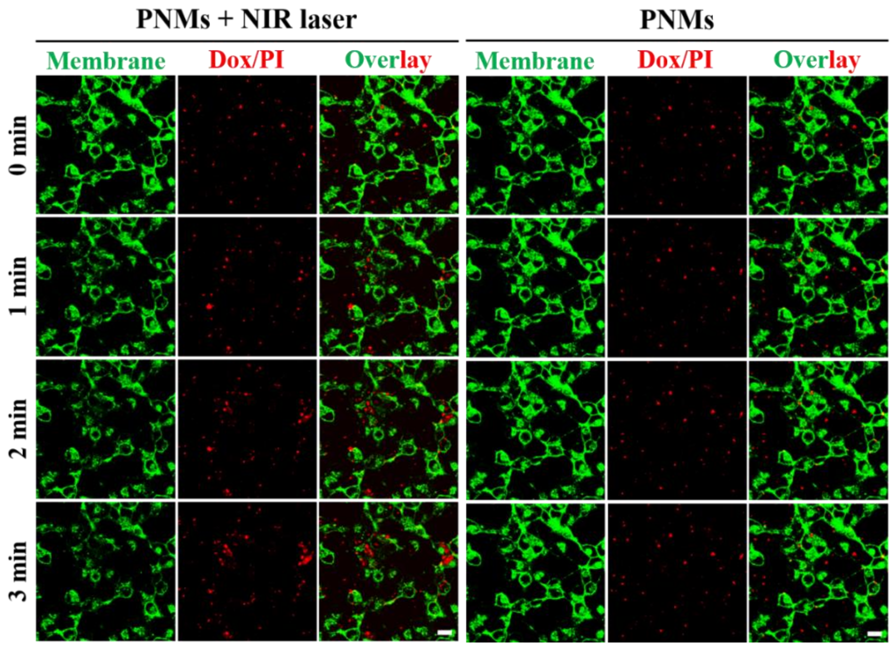

Figure S10. Wide field time-lapsed CLSM images of HeLa cells in presence of PNMs and PI under NIR laser irradiation (left) and without NIR irradiation (right). Scale bar = 20 μm.

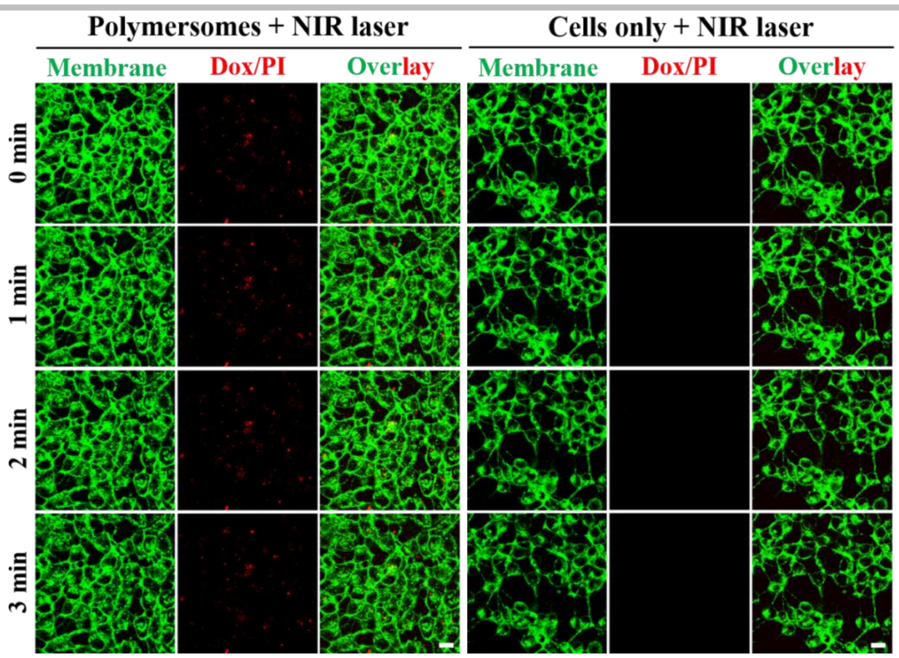

Figure S11. Time-lapsed CLSM images of HeLa cells in presence (left) / absence (right) of polymersomes (without Au coat) under NIR irradiation. PI was introduced to cell medium immediately before CLSM measurement. Scale bar = 20 μm.

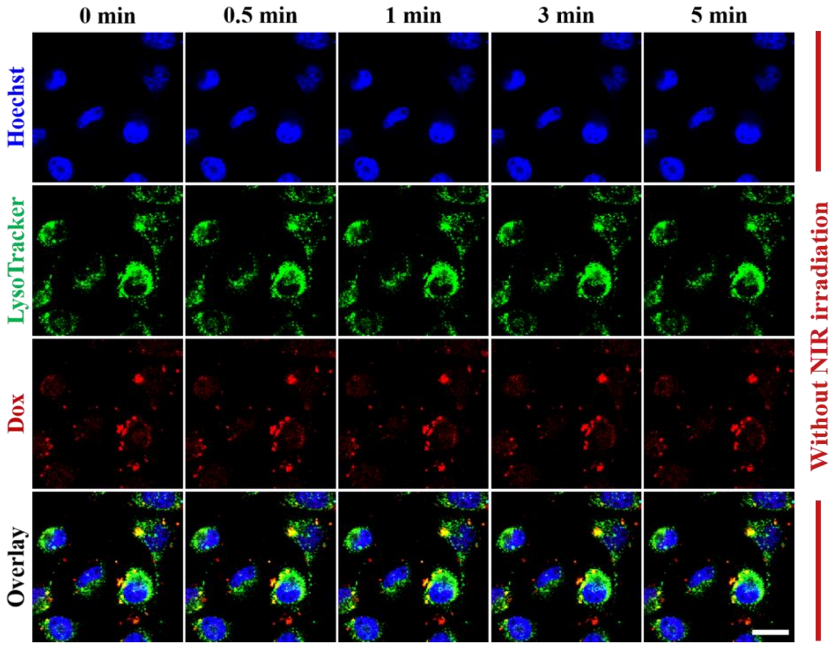

Figure S12. Intracellular localization of PNMs after 4 h of co-culture with HeLa cells in the absence of NIR irradiation. Specific fluorescent probes LysoTracker™ Green DND-26 (Green), Hoechst 33342 (blue), and Dox-PNMs (red) were used to localize lysosomes, cell nucleus, and PNMs, respectively. Scale bar = 20 μm.

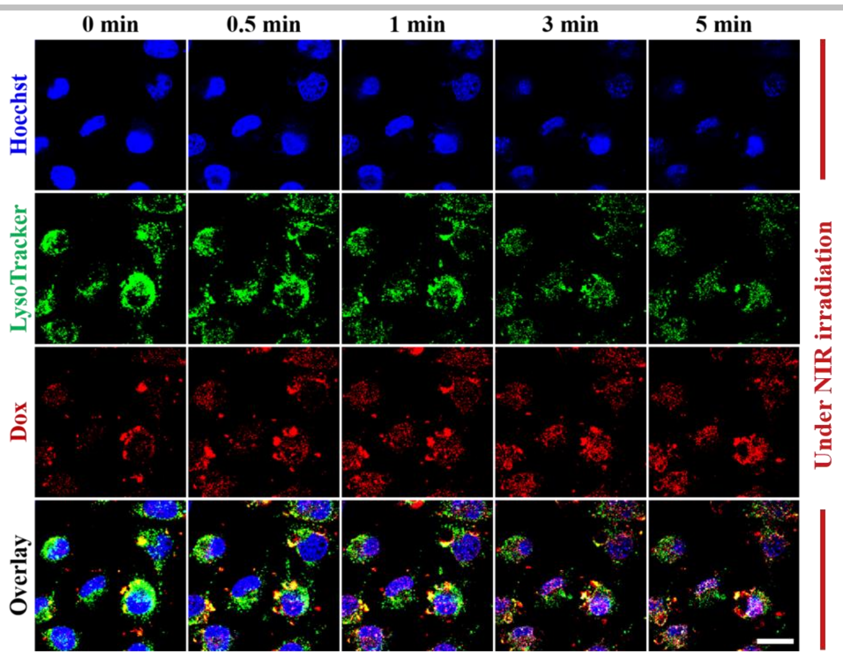

Figure S13. Intracellular localization of PNMs after 4 h of co-culture with HeLa cells under NIR irradiation. Scale bar = 20 μm.

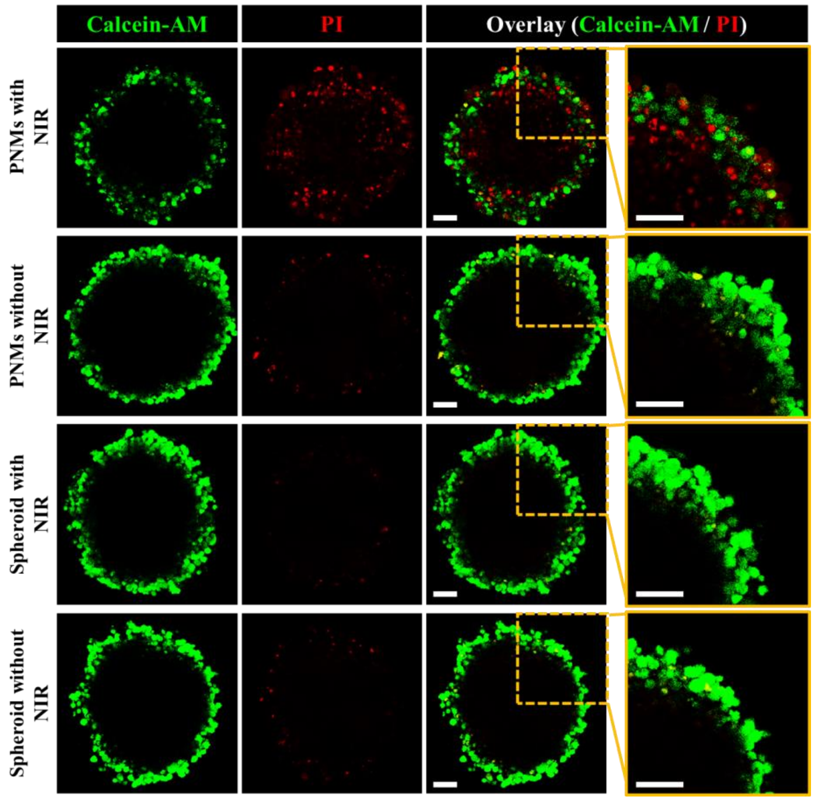

Figure S14. Therapeutic evaluation of PNMs towards 3D HeLa spheroids via live/dead staining images. Scale bar = 50 μm.

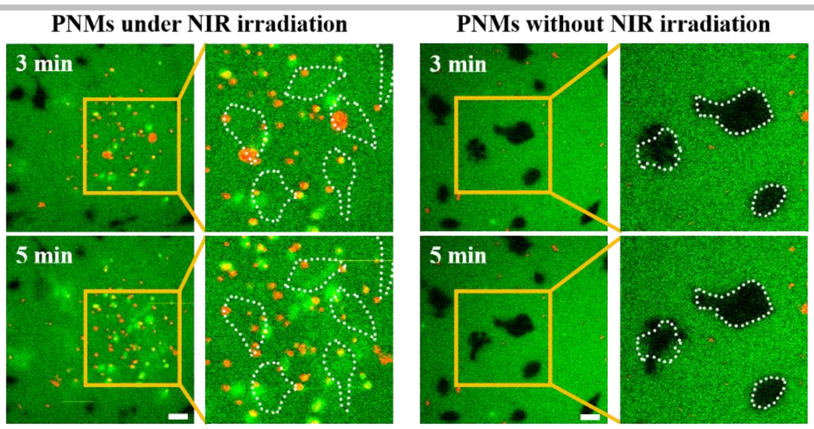

Figure S15. Time-lapsed TP-CLSM images of HeLa cells in presence of FITC-BSA and PNMs with (left) and without (right) NIR laser irradiation. Scale bar = 20 μm.

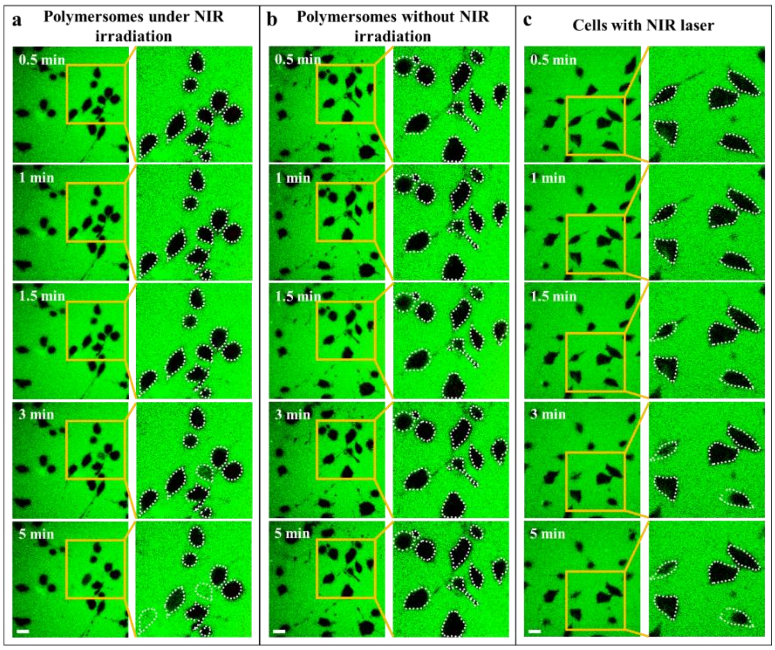

Figure S16. Intracellular delivery of FITC-BSA. FITC-BSA was introduced into the cell medium right before the TP-CLSM measurement. TP-CLSM images of HeLa cells in presence of polymersomes (without Au coat) under NIR laser irradiation (0.8 J cm^{-2}) (a) and without NIR irradiation (b). (c) HeLa cells directly exposed to NIR laser irradiation (0.8 J cm^{-2}). Scale bar = 20 μm.

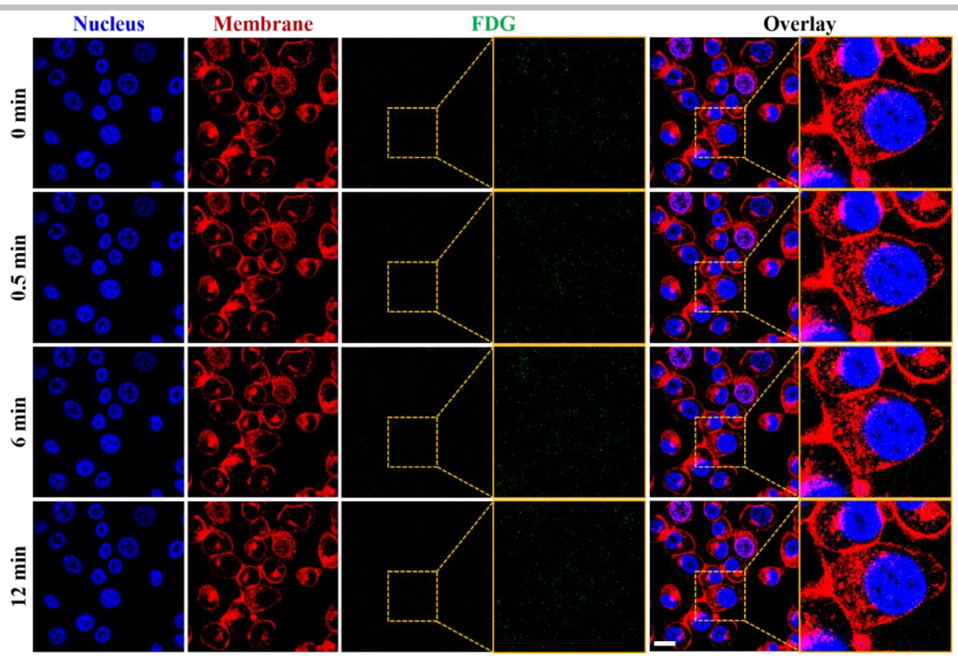

Figure S17. Time-lapsed CLSM images of PNMs-mediated intracellular delivery of FDG and β-gal in the absence of NIR irradiation. Scale bar = 20 μm.

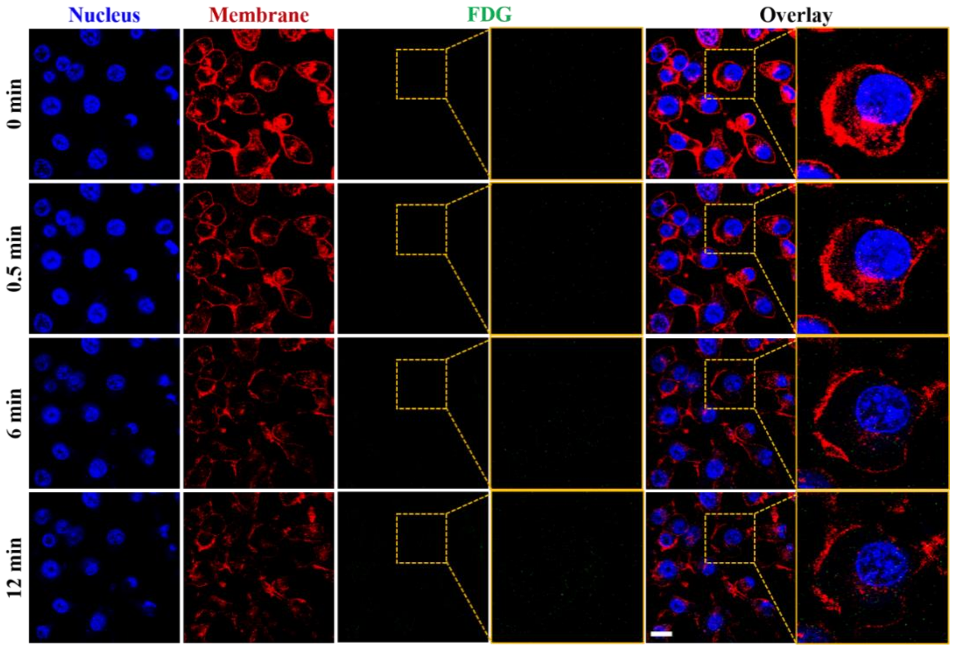

Figure S18. Time-lapsed CLSM images of HeLa cells directly exposed to NIR laser irradiation. Scale bar = 20 μm.

References

[1] X. W. Guan, Z. P. Guo, L. Lin, J. Chen, H. Y. Tian, X. S. Chen, *Nano Lett.* **2016**, *16*, 6823-6831.

[2] N. J. Song, M. M. Ding, Z. C. Pan, J. H. Li, L. J. Zhou, H. Tan, Q. Fu, *Biomacromolecules* **2013**, *14*, 4407-4419.

[3] L. K. E. A. Abdelmohsen, D. S. Williams, J. Pille, S. G. Ozel, R. S. M. Rikken, D. A. Wilson, J. C. M. van Hest, *J. Am. Chem. Soc.* **2016**, *138*, 9353-9356.

[4] Y. J. Wu, T. Y. Si, J. X. Shao, Z. G. Wu, Q. He, *Nano Res.* **2016**, *9*, 3747-3756.

[5] X. Ma, K. Hahn, S. Sanchez, *J. Am. Chem. Soc.* **2015**, *137*, 4976-4979.

[6] a) H. L. Che, J. Z. Zhu, S. D. Song, A. F. Mason, S. P. Cao, I. A. B. Pijpers, L. K. E. A. Abdelmohsen, J. C. M. van Hest, *Angew. Chem. Int. Ed.* **2019**, *58*, 13113-13118; b) I. A. B. Pijpers, S. P. Cao, A. Llopis-Lorente, J. Z. Zhu, S. D. Song, R. R. M. Joosten, F. H. Meng, H. Friedrich, D. S. Williams, S. Sánchez, J. C. M. van Hest, L. K. E. A. Abdelmohsen, *Nano Lett.* **2020**, 20, 4472-4480.

[7] G. Lazzari, P. Couvreur, S. Mura, *Polym. Chem.* **2017**, *8*, 4947-4969.

[8] a) S. Sant, P. A. Johnston, *Drug Discovery Today: Technol.* **2017**, *23*, 27-36; b) J. Friedrich, C. Seidel, R. Ebner, L. A. Kunz-Schughart, *Nat. Protoc.* **2009**, *4*, 309-324; c) D. L. Priwitaningrum, J. G. Blondé, A. Sridhar, J. van Baarlen, W. E. Hennink, G. Storm, S. L. Gac, J. Prakash, *J. Controlled Release* **2016**,

244, 257-268; d) S. P. Cao, L. K. E. A. Abdelmohsen, J. X. Shao, J. van den Dikkenberg, E. Mastrobattista, D. S. Williams, J. C. M. van Hest, *ACS Macro Lett.* **2018**, *7*, 1394-1399.

6. Supplementary videos

Video S1. Nanoparticle tracking video of PNMs (5.19×10^7 particles mL^{-1}) in the absence of NIR laser irradiation
Video S2. Nanoparticle tracking video of PNMs (1.41×10^8 particles mL^{-1}) under NIR laser irradiation (1 W)
Video S3. Nanoparticle tracking video of gold nanoshells (2.89×10^8 particles mL^{-1}) without NIR laser irradiation
Video S4. Nanoparticle tracking video of gold nanoshells (5.11×10^8 particles mL^{-1}) under NIR irradiation (1 W)
Video S5. Motion behavior of polymersomes (without gold coat, 3.89×10^8 particles mL^{-1}) under NIR irradiation (1 W)
Video S6. Intracellular delivery of FITC-BSA (10 μg μL^{-1}) via PNMs under NIR irradiation (0.8 J cm^{-2}, ca. 10 min)
Video S7. Co-introduction of polymersomes and FITC-BSA (10 μg μL^{-1}) to HeLa cells under NIR irradiation (0.8 J cm^{-2}, ca. 10 min)
Video S8. Irradiation of HeLa cells with NIR laser (0.8 J cm^{-2}, ca. 10 min)
Video S9. Intracellular delivery of FITC-BSA (10 μg μL^{-1}) via PNMs without NIR irradiation (ca. 10 min)
Video S10. Co-introduction of polymersomes and FITC-BSA (10 μg μL^{-1}) to HeLa cells without NIR irradiation (ca. 10 min)
Video S11. Co-delivery of enzyme substrates (FDG) and enzyme (β−gal) via PNMs under NIR irradiation (0.1 J cm^{-2}, ca.15 min)

Author Contributions

J. Shao and S. Cao are contributed equally to this work. J. Shao and S. Cao designed and performed the experimental parts, made data analysis. J. Shao wrote the original draft. Dr. D. S. Williams, Dr. L. K. E. A. Abdelmohsen and Prof. J. C. M. van Hest advised and revised the manuscript. Prof. J. C. M. van Hest designed the experimental parts, provided funding, supervised the project. All the authors discussed the results.
